# Dietary Intakes Among University Students in Iceland: Insights from the FINESCOP Project

**DOI:** 10.3390/nu17030432

**Published:** 2025-01-24

**Authors:** Brittany M. Repella, Greta Jakobsdottir

**Affiliations:** Faculty of Health Promotion, Sport and Leisure Studies, School of Education, University of Iceland, 105 Reykjavik, Iceland; brittany@hi.is

**Keywords:** health promotion, nutrition, diet, food choice, dietary patterns

## Abstract

**Objectives**: Using data from the Food Insecurity among European University Students during the COVID-19 Pandemic (FINESCOP) project, this study aims to investigate the dietary intakes among university students in Iceland, focusing specifically on their current diet after March 2020. Additionally, it examines correlations among different food groups to reveal associations in dietary patterns. **Methods**: The investigation uses data from the observational FINESCOP project in Iceland. Spearman’s correlation coefficients were used to identify associations between different dietary intakes. *p* values show significance at a level of <0.05. **Results**: Icelandic university students have a low intake of vegetables and salad (“3–4 times per week”, n = 159, 24.8%), fruit (“1–2 times per week”, n = 164, 25.6%), and whole wheat (“3–4 times per week”, n = 147, 23.2%). Lower than these was legume intake (“Never/seldom”, n = 203, 32.0%). Meat and eggs were consumed more frequently (“3–4 times per week”, n = 231, 36.3%) compared to fish and seafood (“1–2 times per week”, n = 277, 43.5%). While the findings do indicate a significant correlation between vegetables and salad and fruit intake (*p* < 0.001) and between vegetable and salad and legume intake (*p* < 0.001), causal relationships cannot be established. Among discretionary foods, sweets and snacks were moderately and significantly correlated (*p* < 0.001). **Conclusions**: This study explores dietary intakes and correlations between dietary factors among university students in Iceland. Further research is needed to explore the potential for causal inferences and better understand these dietary behaviors of university students in Iceland.

## 1. Introduction

What university students eat is important for their mental and physical health. Among university students in Iceland, 17% (n = 125) experienced some level of food insecurity and nearly 4% (n = 28) went an entire day without eating due to the lack of food or other resources to purchase food [1]. Food intake has been associated with university students’ ability to function as well as their ability to concentrate and to properly study and absorb information [2,3]. Students must be able to consume enough nourishing food throughout the day regardless of their socioeconomic status. The following research looks deeper into what university students in Iceland are eating, and which foods are correlated. The first investigation in Iceland was published in October 2023 and looked at the prevalence of food insecurity and associations with academic performance, consumption patterns, and social support among university students during the COVID-19 pandemic [1].

The Nordic Nutrition Recommendations, which guide the national Icelandic recommendations, are a set of nutritional and health promoting guidelines that are evidence-based. The current guidelines also consider the environment, with an emphasis on plant-based foods [4]. Plant foods, namely vegetables and fruits, are crucial for human health and are the basis of nearly all national dietary recommendations around the world, including the recommendations put forth by the World Health Organization [5]. In addition to the right quantities of food, everyone, including university students, benefits from consuming nutritious foods. Higher intake of plants, like vegetables, fruits, and legumes, can reduce a person’s risk of many noncommunicable diseases, for instance, cardiovascular disease and certain cancers [6,7], and reduce all-cause mortality [7,8].

In Iceland, the dietary recommendations advise adults from the age of two and older to consume five servings of fruits and vegetables, with three of these preferably being a vegetable, whole grains two times a day, fish two to three times a week, a reasonable amount of dairy and dairy products, about twice a day, and a moderate amount of meat, limiting red meat to around two servings a week. Legume intake is recommended at a few times a week. While there is no intake level, the recommendations promote limiting processed foods such as sugar-sweetened beverages, salty snacks, sweets, and processed meat [9]. Even with the national guidelines, Icelanders fall short to reach the intake of plant foods. In the most recent national dietary survey of Icelanders, “What are Icelanders Eating (Hvað borða Íslendingar)” [10], only about 2% of the respondents consumed the recommended five servings (500 g) of vegetables and fruit per day. Iceland falls far behind the consumption range in the European Union, which has an average five serving intake of 12%, ranging from 20% (France) to 33% (Ireland) [11,12].

Legumes are also an important part of the diet and have been linked to reduced noncommunicable diseases and reduced all-cause mortality [13]. As seen in the Icelandic national dietary survey, only 17% of respondents ate beans or lentils and 25% ate a plant-based meal as the main course once or more during the week [10]. While the Nordic Dietary Recommendations are promoting an increase in plant-based foods, especially protein-rich plant foods such as beans and lentils, consuming protein from fish and seafood is also important. Fish and seafood, especially fatty fish, have been linked to an improvement in cardiovascular health [14], mostly due to the omega fatty acids in fatty fish like salmon or cod liver oil [15]. Meat and eggs can also be part of a balanced diet. Most guidelines, including the Nordic and Icelandic, recommend meat in moderation and priority should be placed on leaner cuts of meat, such as chicken and turkey.

The Food Insecurity among European University Students during the COVID-19 Pandemic (FINESCOP) is a cross-sectional investigation of university students, with data collected throughout Europe, such as Iceland, Norway, Finland, Germany, Poland, the Netherlands, Belgium, Portugal, Spain, and Italy. The primary focus of this paper is to analyze dietary intakes of university students in Iceland. The Icelandic dietary guidelines are used for comparisons and to identify specific food groups that are over- or under-consumed relative to the recommendations. Additionally, correlations between foods and food groups are explored.

## 2. Materials and Methods

### 2.1. Study Design

The data for this research come from the FINESCOP project. The following dissemination refers to Icelandic data only, which were collected from the 11th of January until the 31st of March 2022 [1].

### 2.2. Participant Recruitment and Enrollment

Information on participants and enrollment can be found in more detail in the first Icelandic publication about FINESCOP [1]. Eligibility criteria included being a university student at one of the participating universities, aged 18 years or older, and having access to their university email.

### 2.3. Questionnaire Development

In collaboration with participating FINESCOP countries, the questionnaire was developed. Further insights and details can be seen in previously published FINESCOP papers [1,16].

### 2.4. Dietary Intakes Questions

The following food groups were in the FINESCOP questionnaire: vegetables and salad [(juice and potatoes are not included) (one plate, 1–2 cups or 150–200 g each time)]; fruit [(juice not included) (one large piece or two small ones, 1 cup or 120–200 g each time)]; legumes (beans and lentils) [(4 tablespoons of cooked beans, about 60–80 g)]; dairy and dairy products [(one glass or container, about 200–250 g)]; whole wheat products [bowl of cereal, 1 slice of bread, 3 tablespoons of pasta/rice (about 60–80 g each)]; fish and seafood (one portion, about 125–150 g each); meat and eggs [unprocessed products (one portion, about 100–125 g each)]; processed meat [e.g., sausages, ham, bacon (about 100–125 g each)]; salty snacks [salt sticks and flakes (one small bag, about 50–100 g each time)]; sweets [chocolate, biscuits, muffins, pastries, ice cream, and candy (one medium-sized piece, about 50–100 g each)]; sugary drinks/soda [other than energy drinks (one can, about 330 mL each time)]; sugar-free soda [other than energy drinks (one can, about 330 mL each)]; energy drinks (one can, about 330 mL each). Responses included rarely/never; less than once a week; 1–2 times a week; 3–4 times a week; 5–6 times a week; 1 time a day; 2 times a day; 3 times or more a day; don’t know.

Dietary data were collected twice in the questionnaire and the following questions were included: (1) “Before the COVID-19 pandemic (March 2019-March 2020), how many times a week on average did you eat/drink…” and (2) “Since the COVID-19 epidemic started, in March 2020, how many times a week on average did you eat/drink…”. For this study, we focus only on the diet after March 2020, or the current diet of the respondents, and do not include any pre-pandemic data. Dietary intake descriptives are shown as both the number of respondents and the percentage of the total and cumulative percentage.

When making statements about correct dietary intakes, the researchers use and refer to either the Icelandic dietary recommendations or the Nordic recommendations [4,9]. Since Iceland bases its recommendations on the Nordic recommendations, for the sake of brevity, all statements will be referred to as dietary recommendations.

### 2.5. Data Analysis

Descriptives and analysis were completed using RStudio v4.6.4 (R Core Team 2022) and jamovi (Version 2.5).

A correlation matrix was created with the dietary intake variables, and Spearman’s correlation coefficients were used to assess how well the relationship is between the variables. Spearman’s rank correlation was chosen due to the responses in the dietary questions, which are measured using an ordinal scale. A positive correlation suggests that both variables move in the same direction, while a negative correlation suggests that variables move in the opposite direction. The closer the correlation comes to one (±), the stronger the correlation is. The correlation ranges include Spearman’s rho (ρ) = ±1 (perfect correlation), ±0.7 < ρ < ±1 (strong correlation), ±0.4 < ρ ≤ ±0.7 (moderate correlation), and 0 < ρ ≤ ±0.4 (weak correlation). Additionally, the correlation can be non-existent or zero, ρ = 0, which implies no relationship between the two variables.

### 2.6. Ethical Consideration

This study complied with ethical standards aimed at protecting participant confidentiality and rights. Each participant completed a digital consent form before taking part in the survey, and participation was contingent upon receiving this consent. The institutional review board of the university’s Ethics Committee for Scientific Research reviewed and approved this study (SHV2021-038).

## 3. Results

### 3.1. Respondent Demographics

The university cohort’s demographics can be seen in the first published paper from Repella et al. [1]. The students were mostly female (74.5%) originating in Iceland (78.5%). The average age of the respondents was 31.7 years (SD 8.4).

### 3.2. Dietary Intakes

Table 1 shows dietary intakes. The dietary recommended level will be in bold text if applicable and as accurately as possible. Since vegetables and salad and fruit are combined in the dietary recommendations as five per day, more than one intake level will be bold in fruit.

For the food groups that have a recommendation in Iceland, the respondent’s dietary intake fell short. For vegetables and salad, just 15 respondents (2.3%) reached the “3 times or more per day” recommended intake level. Fruit was also low, with only 46 respondents (7.2%) reaching the recommended “2 times per day”. Intake of whole wheat had just 50 respondents (7.9%) and dairy and dairy products had 49 respondents (7.7%) reach the recommended intake. Nearly half of the respondents consumed fish and seafood “1–2 times per week” (n = 277, 43.5%) and meat and eggs at a higher frequency (“3–4 times per week”) but with slightly fewer respondents (n = 231, 36.3%).

Salty snacks were consumed “1–2 times per week” (n = 198, 30.9%) and sweets were consumed slightly more frequently at “3–4 times per week” (n = 180, 28.1%). About three-quarters of the respondents consumed processed meat less than two times per week, split nearly evenly among the three frequency groups: “1–2 times per week” (n = 164, 25.8%), “less than once a week” (n = 163, 25.7%), and “never/seldom” (n = 164, 25.8%). When looking at beverages, the highest intake group, “never/seldom”, was the same for sugar-sweetened beverages, sugar-free beverages, and energy drinks, as seen in Table 1.

### 3.3. Correlation and Association

A correlation matrix was created for the food recommended by the dietary recommendations: vegetables and salad, fruit, fish and seafood, meat and eggs, legumes, dairy and dairy products, and whole wheat products, as can be seen in Table 2. The strength of correlations is categorized based on Spearman’s coefficient thresholds [17]. Fifteen significant correlations were found, though the strength of the correlations was weak. One strong positive correlation was found between the intake of vegetables and salad and fruit (Spearman’s rho = 0.647, *p* < 0.001). Another significant correlation was found between the intake of vegetables and salad and legumes, moderately positive (Spearman’s rho = 0.403, *p* < 0.001).

A correlation matrix was also created for processed foods and drinks with the assumption that all correlations would be positive: salty snacks, sweets, sugar drinks and soda, sugar-free soda, energy drinks, and processed meat, as shown in Table 3. Among these pairs, only one was ranked moderately positive, salty snacks and sweets (Spearman’s rho = 0.405, *p* < 0.001), while the remaining pairs were significant, but with a weak correlation strength.

## 4. Discussion

The purpose of this research was to describe the dietary intake of university students in Iceland and identify correlations among dietary intake variables from the FINESCOP questionnaire. While other variables were measured in the questionnaire, this research looks only at dietary intake and only after the COVID-19 pandemic and, therefore, the current diet of university students in Iceland. For results on other variables from the Icelandic study, see the first paper by Repella et al. [1].

The dietary guidelines in Iceland recommend five servings of vegetables and fruits per day, with the idea that more than half of them comprise vegetables. According to the most recent Icelandic national dietary survey, “What are Icelanders Eating (Hvað borða Íslendingar)“ [10], only about 2% of respondents reached the recommended intake level of five servings per day of vegetables and fruit, or about 500 g. The average intake was about 213 g, just slightly under half of the recommendations [9]. The current research shows similar failings to reach the recommended amount, with 24.8% (n = 159) respondents consuming vegetables three to four times per week, far from three times per day. For fruit intake, only 7.2% (n = 46) reached the recommendation, while the greatest number of respondents consumed fruit one to two times per week, 25.6% (n = 164).

The current FINESCOP questionnaire kept vegetables and fruit separate. To make the comparison possible, the minimum intake of three vegetables and two to three fruits per day were combined to create a vegetable and fruit intake grouping. This revealed that 11.1% (n = 71) met the recommended minimum of five servings of produce per day. This low percentage, only 11%, is not ideal for the health of university students. Vegetables and fruit should be the basis of the diet, and they provide many necessary vitamins and minerals. The intake of vegetables and fruits has been linked to concentration improvements in university students [3], in addition to better mood and lower depression [18]. While the percentage calculated from the Icelandic FINESCOP cohort is much higher than that of the national survey results, it is close to the EU average of 12% [11,12]. However, both percentages still reveal a low intake of fruits and vegetables. This could be due to many reasons such as time restrictions, convenience, taste, and knowledge of the benefits of consuming these foods. The exact reason as to why both Iceland and the EU have low intake of vegetables and fruit is unknown; therefore, more research into the exact reasons is needed.

Concerning other food groups, 43.5% (n = 277) of respondents consumed fish and seafood “1–2 times per week” and 72 respondents (11.3%) consumed it “3–4 times per week”. This falls in line with the recommended intake of several times per week, a vague amount that lets the person decide for themselves. The consumption of dairy and dairy products also comes close to the dietary recommendations (two times per day), with the most respondents consuming dairy and dairy products “once per day” (n = 122, 19.3%). Meat and eggs, which do not specify the type of meat, such as white or dark meat, were consumed “3–4 times per week” by the greatest number of respondents (n = 231, 36.3%). This does follow the national dietary guidelines, which recommend meat in moderation with red meat consumption not exceeding 500 g per week. Legumes and whole wheat products are the furthest from the recommendations, with 32.0% (n = 203) consuming legumes “never/seldom” and 23.2% (n = 147) consuming whole wheat “3–4 times per week”, rather than a few times a week and twice per day, respectively.

For processed foods, such as processed meat, salty snacks, sweets, sugary drinks/soda, sugar-free soda, and energy drinks, the respondents follow dietary recommendations of limiting consumption to less than four times per week. Intake of processed meat showed an interesting response split, with nearly a triple tie at about 25% each over three frequencies. After combining the frequencies, just around 75% of respondents consumed processed meat no more than two times per week (77.3%, n = 491). Processed meats have been linked to many chronic diseases, such as heart disease and certain cancers [19,20] and most national dietary recommendations, including those from Iceland, recommend people consume as little as possible [9]. Salty snacks were consumed four or less times a week (76.3%, n = 489) and sweets by slightly fewer respondents (65.4%, n = 419). These two food groups were also moderately positively correlated with each other (Spearman’s rho = 0.405, *p* < 0.001), suggesting that as snack consumption increases, the consumption of sweets tends to increase as well. However, it is important to note that this association is not perfectly strong, so other factors may also be influencing this relationship and further investigation is needed. These results are not similar to what other research shows on university students’ processed food habits, which shows university students consume a large part of their daily diet from processed foods [21,22]. Processed foods, such as salty snacks and sweets, have been associated with increased BMI and an increased risk all-cause mortality [22,23]. Why this is different within the Icelandic population is not known; therefore, further dietary intake research can benefit this body of knowledge.

Concerning how other variables were associated, there was a strong positive correlation between vegetable and salad intake and fruit intake (Spearman’s rho = 0.647, *p* < 0.001) and a moderate positive correlation between vegetable and salad intake and legumes (Spearman’s rho = 0.403, *p* < 0.001), which indicates that as university students increase their consumption of vegetables and salads, they are also likely to increase their fruit intake, and vice versa; similarly, if their vegetable and salad consumption decreases, their fruit intake is likely to decrease as well. While the intake of vegetables and salad and fruit was low (frequency), it may be that those who choose healthy foods, such as vegetables, will also choose other healthy ingredients, such as fruit [21,22,24]. While further analysis is needed to show and further confirm causality, it may benefit students for the university commissaries to promote these foods together. Interestingly, there were no correlations found between meat and eggs and vegetables and salad or with fruit. There was a significant, but weak, negative correlation between meat and eggs and legumes (Spearman’s rho = −0.280, *p* < 0.001); however, without knowing more about the respondent’s diet, such as if they follow a vegetarian or vegan food pattern, it is not possible to infer why these correlations occur.

The reasons behind food purchases and preferences among university students have been shown to be mostly taste, value, convenience, and cost [25,26]. A Dutch study looked at the effects of a conveniently located and free farm stand in university buildings and its effects on the intake of produce. The study revealed an increase in both vegetables and fruit, especially among those who had a lower intake prior to the farm stand being set up [27]. Any approach which promotes an increased intake of recommended food groups helps to improve health and well-being [28]. By having more offerings at school, in and around campus buildings, hopefully the intake of these important food groups will increase.

The findings from this study are important for understanding the dietary habits of university students in Iceland. When comparing the dietary intakes to the recommended dietary intakes, the diet of university students in Iceland falls short. The most recent report of dietary intake among people residing in Iceland also found the diet of the general population to have trouble reaching the national dietary recommendations. First and foremost, for both university students and the public, education can be used to promote healthier diets. Food and nutrition education may increase cooking self-efficacy, the use of vegetables and fruits, and behavioral outcomes, such as increased fruit and vegetable consumption [1,29]. Therefore, it is important to promote education, specifically nutrition education, for all students.

Further research must be conducted to explain the causal relationship between vegetables and fruit. Correlation does not imply causation, but it does indicate associations, which can be useful when planning promotions for dietary intakes, as seen in the Dutch food stand study [27]. FINESCOP was not designed to collect comprehensive dietary intake data [1]. The results from Iceland (n = 924) do represent a sample of university students in Iceland, a strength of this study. The sample largely represents the student population, as seen in the ratio of male and female students at the University of Iceland, approximately 32% and 68% in 2022, respectively, which is similar to, but not exactly the same as, our respondents, 22% male and 75% female [1,30]. According to Statistics Iceland, in 2022, 45% of university students were between 18 and 25 years old and 55% were aged 26 and older. While we had fewer students aged 18–25 (29%), our study also had a high number of students aged 26 and older (71%) [31]. Ph.D. students were also included in our research, which may have brought the students’ age up. The general academic spread was also comparable to our respondents, with 53% being undergraduates and 43% being either postgraduate or other compared to the universities’ figures, which show 57% undergraduates and 42% postgraduate or other [1,32]. A limitation of the research includes a low response rate (4.4%), which may have been due to the need to advertise the questionnaire more. Additionally, the dietary data were self-reported; therefore, bias may have occurred, such as under- or over-reporting and recall bias. The findings are limited to university students.

## 5. Conclusions

The overall dietary intake of recommended foods among these university respondents is generally low in comparison to the dietary recommendations in Iceland, except for fish and seafood and meat and eggs. The significant correlations between dietary behaviors suggest a pattern of eating, which can be used to promote healthier foods to university students. Future research would be valuable in establishing whether increased vegetable and salad intake directly causes an increase in intake of fruit and legumes and how we can use this knowledge to increase healthy patterns of eating in general. Therefore, a longitudinal study design with a food frequency questionnaire is recommended.

## Figures and Tables

**Table 1:** Dietary intakes of university students in Iceland.

	Counts		% of Total		Cumulative %	
Vegetable and Salad						
**3 times or more per day**	**15**		**2.3%**		**2.3%**	
2 times per day	69		10.8%		13.1%	
Once per day	112		17.5%		30.6%	
5–6 times per week	101		15.8%		46.3%	
3–4 times per week	159		24.8%		71.1%	
1–2 times per week	111		17.3%		88.5%	
Less than once a week	41		6.4%		94.9%	
Never/seldom	29		4.5%		99.4%	
Don’t know	4		0.6%		100.0%	
Fruit						
**3 times or more per day**	**22**		**3.4%**		**3.4%**	
**2 times per day**	**46**		**7.2%**		**10.6%**	
Once per day	86		13.4%		24.0%	
5–6 times per week	73		11.4%		35.4%	
3–4 times per week	128		20.0%		55.4%	
1–2 times per week	164		25.6%		81.0%	
Less than once a week	90		14.0%		95.0%	
Never/seldom	28		4.4%		99.4%	
Don’t know	4		0.6%		100.0%	
Legumes						
3 times or more per day	3		0.5%		0.5%	
2 times per day	3		0.5%		0.9%	
Once per day	15		2.4%		3.3%	
5–6 times per week	21		3.3%		6.6%	
**3–4 times per week**	**68**		**10.7%**		**17.4%**	
1–2 times per week	120		18.9%		36.3%	
Less than once a week	179		28.2%		64.5%	
Never/seldom	203		32.0%		96.5%	
Don’t know	22		3.5%		100.0%	
Whole Wheat						
3 times or more per day	13		2.1%		2.1%	
**2 times per day**	**50**		**7.9%**		**9.9%**	
Once per day	132		20.8%		30.8%	
5–6 times per week	110		17.4%		48.1%	
3–4 times per week	147		23.2%		71.3%	
1–2 times per week	95		15.0%		86.3%	
Less than once a week	48		7.6%		93.8%	
Never/seldom	39		6.2%		100.0%	
Don’t know	0		0		0	
Dairy and Dairy Products						
3 times or more per day	11		1.7%		1.7%	
**2 times per day**	**49**		**7.7%**		**9.5%**	
Once per day	122		19.3%		28.8%	
5–6 times per week	71		11.2%		40.0%	
3–4 times per week	114		18.0%		58.0%	
1–2 times per week	103		16.3%		74.2%	
Less than once a week	70		11.1%		85.3%	
Never/seldom	91		14.4%		99.7%	
Don’t know	2		0.3%		100.0%	
Fish and Seafood						
3 times or more per day	0		0		0	
2 times per day	0		0		0	
Once per day	3		0.5%		0.5%	
5–6 times per week	4		0.6%		1.1%	
**3–4 times per week**	**72**		**11.3%**		**12.4%**	
**1–2 times per week**	**277**		**43.5%**		**55.9%**	
Less than once a week	167		26.2%		82.1%	
Never/seldom	112		17.6%		99.7%	
Don’t know	2		0.3%		100.0%	
Meat and Eggs						
3 times or more per day	0		0		0	
2 times per day	9		1.4%		1.4%	
Once per day	41		6.4%		7.8%	
5–6 times per week	88		13.8%		21.7%	
**3–4 times per week**	**231**		**36.3%**		**57.9%**	
**1–2 times per week**	**144**		**22.6%**		**80.5%**	
Less than once a week	41		6.4%		87.0%	
Never/seldom	80		12.6%		99.5%	
Don’t know	3		0.5%		100.0%	
Salty Snacks						
3 times or more per day	4		0.6%		0.6%	
2 times per day	6		0.9%		1.6%	
Once per day	18		2.8%		4.4%	
5–6 times per week	49		7.6%		12.0%	
3–4 times per week	117		18.3%		30.3%	
**1–2 times per week**	**198**		**30.9%**		**61.2%**	
Less than once a week	174		27.1%		88.3%	
Never/seldom	71		11.1%		99.4%	
Don’t know	4		0.6%		100.0%	
Sweets						
3 times or more per day	12		1.9%		1.9%	
2 times per day	20		3.1%		5.0%	
Once per day	63		9.8%		14.8%	
5–6 times per week	94		14.7%		29.5%	
3–4 times per week	180		28.1%		57.7%	
**1–2 times per week**	**139**		**21.7%**		**79.4%**	
Less than once a week	100		15.6%		95.0%	
Never/seldom	31		4.8%		99.8%	
Don’t know	1		0.2%		100.0%	
Sugar Drinks and Soda						
3 times or more per day	5		0.8%		0.8%	
2 times per day	5		0.8%		1.6%	
Once per day	19		3.0%		4.5%	
5–6 times per week	14		2.2%		6.7%	
3–4 times per week	51		8.0%		14.7%	
**1–2 times per week**	**97**		**15.2%**		**29.9%**	
Less than once a week	139		21.8%		51.6%	
Never/seldom	309		48.4%		100.0%	
Don’t know	0		0		0	
Processed Meat						
3 times or more per day	0		0		0	
2 times per day	2		0.3%		0.3%	
Once per day	15		2.4%		2.7%	
5–6 times per week	25		3.9%		6.6%	
3–4 times per week	100		15.7%		22.4%	
1–2 times per week	164		25.8%		48.2%	
Less than once a week	163		25.7%		73.9%	
Never/seldom	164		25.8%		99.7%	
Don’t know	2		0.3%		100.0%	
Sugar-Free Soda						
3 times or more per day	23		3.6%		3.6%	
2 times per day	23		3.6%		7.2%	
Once per day	41		6.4%		13.6%	
5–6 times per week	40		6.3%		19.9%	
3–4 times per week	85		13.3%		33.2%	
1–2 times per week	84		13.2%		46.4%	
Less than once a week	84		13.2%		59.6%	
Never/seldom	253		39.7%		99.2%	
Don’t know	5		0.8%		100.0%	
Energy Drinks						
3 times or more per day	7		1.1%		1.1%	
2 times per day	17		2.7%		3.8%	
Once per day	61		9.5%		13.3%	
5–6 times per week	28		4.4%		17.7%	
3–4 times per week	50		7.8%		25.5%	
1–2 times per week	53		8.3%		33.8%	
Less than once a week	80		12.5%		46.3%	
Never/seldom	343		53.7%		100.0%	
Don’t know	0		0		0	

**Table 2:** Correlation matrix of dietary recommended foods.

Variable 1	Variable 2	Spearman’s Rho	Strength	*df* ^1^	*p*-Value
Vegetables and Salad					
	Fruit (n = 637)	0.647 ***	Strong (+)	635	<0.001
	Legumes (n = 612)	0.403 ***	Moderate (+)	610	<0.001
	Whole Wheat (n = 630)	0.279 ***	Weak (+)	628	<0.001
	Dairy and Dairy Products (n = 629)	0.078	Weak (+)	627	0.05
	Fish and Seafood (n = 633)	0.106 **	Weak (+)	631	<0.01
	Meat and Eggs (n = 632)	0.001	No correlation	630	0.972
Fruit					
	Legumes (n = 612)	0.208 ***	Weak (+)	610	<0.001
	Whole Wheat (n = 630)	0.271 ***	Weak (+)	628	<0.001
	Dairy and Dairy Products (n = 629)	0.104 **	Weak (+)	627	<0.01
	Fish and Seafood (n = 633)	0.152 ***	Weak (+)	631	<0.001
	Meat and Eggs (n = 632)	0.009	No correlation	630	0.822
Legumes					
	Whole Wheat (n = 611)	0.175 ***	Weak (+)	609	<0.001
	Dairy and Dairy Products (n = 611)	−0.113 **	Weak (−)	609	0.005
	Fish and Seafood (n = 612)	−0.053	Weak (−)	610	0.189
	Meat and Eggs (n = 611)	−0.280 ***	Weak (−)	609	<0.001
Whole Wheat					
	Dairy and Dairy Products (n = 630)	0.219 ***	Weak (+)	628	<0.001
	Fish and Seafood (n = 632)	−0.056	Weak (−)	630	0.160
	Meat and Eggs (n = 631)	−0.046	Weak (−)	629	0.25
Dairy and Dairy Products					
	Fish and Seafood (n = 631)	0.235 ***	Weak (+)	629	<0.001
	Meat and Eggs (n = 630)	0.214 ***	Weak (+)	628	<0.001
Fish and Seafood					
	Meat and Eggs (n = 634)	0.294 ***	Weak (+)	632	<0.001

^1^ degrees of freedom: *df* = n − 2. Note: ** *p* < 0.01, *** *p* < 0.001, one-tailed.

**Table 3:** Correlation matrix of processed food and drinks.

Variable 1	Variable 2	Spearman’s Rho	Strength	*df* ^1^	*p*-Value
Salty Snacks					
	Sweets (n = 636)	0.405 ***	Moderate (+)	634	<0.001
	Sugar Drinks and Soda (n = 635)	0.234 ***	Weak (+)	633	<0.001
	Processed Meat (n = 631)	0.131 ***	Weak (+)	629	<0.001
	Sugar-Free Soda (n = 629)	0.124 ***	Weak (+)	627	<0.001
	Energy Drinks (n = 635)	0.097 **	Weak (+)	633	<0.01
Sweets					
	Sugar Drinks and Soda (n = 637)	0.209 ***	Weak (+)	635	<0.001
	Processed Meat (n = 632)	0.143 ***	Weak (+)	630	<0.001
	Sugar-Free Soda (n = 631)	0.094 **	Weak (+)	629	<0.01
	Energy Drinks (n = 637)	0.036	No correlation	635	0.179
Sugar Drinks and Soda					
	Processed Meat (n = 633)	0.209 ***	Weak (+)	631	<0.001
	Sugar-Free Soda (n = 633)	−0.035	No correlation	631	0.812
	Energy Drinks (n = 639)	0.071 *	Weak (+)	637	<0.05
Processed Meat					
	Sugar-Free Soda (n = 627)	0.182 ***	Weak (+)	625	<0.001
	Energy Drinks (n = 633)	0.144 ***	Weak (+)	631	<0.001
Sugar-Free Soda					
	Energy Drinks (n = 633)	0.213 ***	Weak (+)	631	<0.001

^1^ degrees of freedom: *df* = n − 2. Note: * *p* < 0.05, ** *p* < 0.01, *** *p* < 0.001, one-tailed.

## Data Availability

The original contributions presented in this study are included in the article. Further inquiries can be directed to the corresponding author.
